# Frequency and predictors of poststroke epilepsy after mechanical thrombectomy for large vessel occlusion stroke: results from a multicenter cohort study

**DOI:** 10.1007/s00415-023-11966-x

**Published:** 2023-09-02

**Authors:** Joachim Gruber, Thomas Gattringer, Georg Mayr, Daniel Schwarzenhofer, Markus Kneihsl, Judith Wagner, Michael Sonnberger, Hannes Deutschmann, Melanie Haidegger, Simon Fandler-Höfler, Stefan Ropele, Christian Enzinger, Tim von Oertzen

**Affiliations:** 1 Department of Neurology 1, Neuromed Campus, Kepler University Hospital, Wagner-Jauregg-Weg 15, 4020 Linz, Austria; 2 Department of Neurology, Medical University of Graz, Auenbruggerplatz 22, 8026 Graz, Austria; 3 Division of Neuroradiology, Vascular and Interventional Radiology, Department of Radiology, Medical University of Graz, Graz, Austria; 4 Department of Neuroradiology, Neuromed Campus, Kepler University Hospital, Linz, Austria; 5 Department of Neurology, Evangelisches Klinikum Gelsenkirchen, Academic Hospital University Essen-Duisburg, Gelsenkirchen, Germany

**Keywords:** Stroke, Mechanical thrombectomy, Endovascular stroke treatment, Epilepsy, Poststroke epilepsy

## Abstract

**Background:**

Poststroke epilepsy (PSE) represents an important complication of stroke. Data regarding the frequency and predictors of PSE in patients with large-vessel occlusion stroke receiving mechanical thrombectomy (MT) are scarce. Furthermore, information on acute and preexisting lesion characteristics on brain MRI has not yet been systematically considered in risk prediction of PSE. This study thus aims to assess PSE risk after acute ischemic stroke treated with MT, based on clinical and MRI features.

**Methods:**

In this multicenter study from two tertiary stroke centers, we included consecutive acute ischemic stroke patients who had received MT for acute intracranial large vessel occlusion (LVO) between 2011 and 2017, in whom post-interventional brain MRI and long term-follow-up data were available. Infarct size, affected cerebrovascular territory, hemorrhagic complications and chronic cerebrovascular disease features were assessed on MRI (blinded to clinical information). The primary outcome was the occurrence of PSE (> 7 days after stroke onset) assessed by systematic follow-up via phone interview or electronic records.

**Results:**

Our final study cohort comprised 348 thrombectomy patients (median age: 67 years, 45% women) with a median long-term follow-up of 78 months (range 0–125). 32 patients (9%) developed PSE after a median of 477 days (range 9–2577 days). In univariable analyses, larger postinterventional infarct size, infarct location in the parietal, frontal or temporal lobes and cerebral microbleeds were associated with PSE. Multivariable Cox regression analysis confirmed larger infarct size (HR 3.49; 95% CI 1.67–7.30) and presence of cerebral microbleeds (HR 2.56; 95% CI 1.18–5.56) as independent predictors of PSE.

**Conclusion:**

In our study, patients with large vessel occlusion stroke receiving MT had a 9% prevalence of PSE over a median follow-up period of 6.5 years. Besides larger infarct size, presence of cerebral microbleeds on brain MRI predicted PSE occurrence.

**Supplementary Information:**

The online version contains supplementary material available at 10.1007/s00415-023-11966-x.

## Introduction

Patients with cerebrovascular disorders have an increased risk of developing seizures, with stroke being the major cause of acquired epilepsy in adults [1]. Poststroke seizures are divided into early versus late seizures (≤ 7 vs. > 7 days after the ictus) [2]. While early seizures represent an acute reaction of neuronal injury and are regarded provoked, late seizures after stroke indicate neuronal hyperexcitability and reorganization of damaged networks and fulfill the diagnosis of post-stroke epilepsy (PSE) with an observed epilepsy risk of 60% in the following 10 years [3]. The incidence of PSE at 5 years has been estimated as 8% for ischemic and 12% in hemorrhagic strokes [4, 5]. Further knowledge on risk prediction of PSE after stroke is of particular importance as PSE is associated with serious complications, such as injuries from seizures, status epilepticus and side effects of antiseizure medication.

The “SeLECT Score” [Severity of stroke (NIHSS score), Large-artery atherosclerotic etiology, Early seizures, Cortical involvement, Territory of MCA] has recently been proposed as an easy-to-use instrument based on five clinical predictors in order to identify individuals at high risk of PSE [5]. However, this tool was developed in a general stroke cohort and not specifically in those who had received acute recanalization therapy.

Most prior studies describing relevant brain imaging risk factors for (late) epileptic seizures after stroke such as cortical involvement, large infarct size, hemorrhagic transformation, cerebral small vessel disease or lower brain volume, were mainly CT-based with decreased sensitivity to detect parenchymal brain abnormalities [6–10]. Further, only few prior studies have investigated specific brain infarct locations as potential epileptogenic regions after ischemic stroke [11–14].

Moreover, there are still uncertainties concerning the relationship between different recanalization therapies (e.g., intravenous thrombolysis (IVT) and/or endovascular mechanical thrombectomy), although there seems to be little impact on the development of epileptic activity after thrombolysis [15–17]. A recent systematic review and meta-analysis on the cumulative incidence of seizures in stroke patients following endovascular treatment suggested 5.8%, but included studies with different inclusion and exclusion criteria, also took patients only treated with IVT into account, had a diverse classification of poststroke epilepsy and an unknown or varying length of follow-up (range 0–5 years) [18].

We here thus sought to overcome these limitations by investigating the potential role of clinical and MRI parameters in predicting the risk of PSE after endovascular stroke therapy for large intracranial artery occlusion.

## Methods

This study was conducted at the Department of Neurology 1, Neuromed Campus, Kepler University Hospital, Linz, Austria and at the Department of Neurology, Medical University of Graz, Austria. We chose a retrospective design with a prospective follow-up. All patients who had been hospitalized due to an acute ischemic stroke from 2011 to 2017 who had undergone mechanical thrombectomy for intracranial large vessel occlusion were included into the study, provided they had a post-interventional brain MRI. Affected intracranial vessels comprised the internal carotid artery, middle cerebral artery, anterior cerebral artery, posterior cerebral artery and the basilar artery.

We excluded patients without a postinterventional brain MRI, younger than 18 years at stroke onset, who had previous epileptic seizures or pre-existing brain lesions and those who died within 24 h of stroke onset.

All patients of the Linz cohort were followed-up with a structured telephone interview based on a validated questionnaire to detect seizures [19]. In participants who did not have the capacity to answer the questionnaire, we interviewed close relatives, nursing staff or their general practitioner. Positive answers resulted in a face-to-face neurological consultation, including a thorough clinical exploration and an electroencephalogram (EEG), to determine the epileptic nature of reported episodes and to minimize the possibility of seizure mimics.

Follow-up information for the Graz cohort was extracted from the fully-electronic medical documentation network connecting all Styrian public hospitals (called MEDOCS). In the province of Styria, specialized acute neurological care including acute epileptic seizures is provided by five public acute-care neurological departments. MEDOCS therefore allows to detect all outpatient hospital visits or hospitalizations—including those due to a new onset seizure [20].

Supplemental Table S1 gives an overview on collected clinical and neuroimaging data.

Postinterventional brain MRI scans (usually on day 1 after thrombectomy) were evaluated by three trained raters (J.G., G.M., M.S.) with neurovascular expertise. MRI protocols included at least one blood-sensitive sequence (either T2* gradient-echo or susceptibility-weighted imaging), T2-weighted fluid attenuated inversion recovery (FLAIR), T2-weighted and diffusion-weighted images (DWI) as well as an intracranial time-of-flight MR-angiography (TOF-MRA).

MRI scans were analyzed regarding pre-defined imaging variables (Supplemental Table S1), raters were blinded to the occurrence of seizures.

For statistical analysis, IBM SPSS Statistics^©^ Version 28 was used. After confirming the proportional hazards assumption, we used Cox regression to estimate hazard ratios of variables potentially associated with our primary outcome variable, occurrence of PSE (> 7 days after stroke onset) in uni- and multivariable analysis. For the multivariable model, we included all variables with a *p*-value of < 0.05 in univariable analysis.

The ethics committees of the Johannes Kepler University Linz and the Medical University of Graz approved the study (Approval No. Linz: 1183/2020, Graz: 32-634 ex 19/20).

## Results

**Fig. 1:**
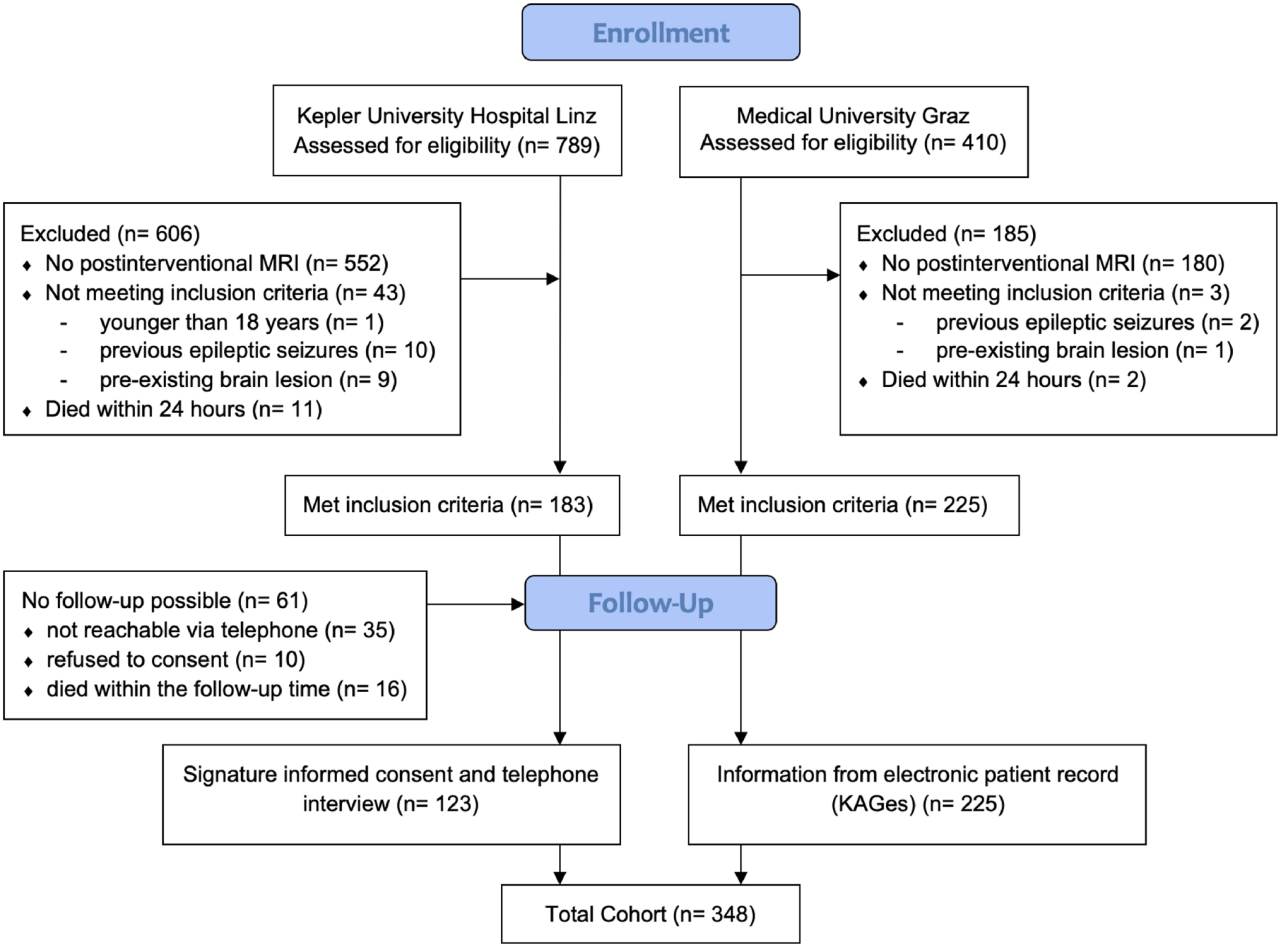
Flow chart of patient selection

1052 patients with ischemic stroke that had received mechanical thrombectomy were retrospectively identified within the study period; 348 patients (median age: 67 years, 45% women, median admission National Institutes of Health Stroke Scale (NIHSS): 15, IV thrombolysis rate: 69.3%) finally fulfilled the inclusion and exclusion criteria as shown in Fig. 1*.* Successful recanalization according to TICI 2b-3 was achieved in 92.5%. Further clinical and neuroimaging information is provided in Tables 1 and 2.

**Table 1:** Demographic and clinical characteristics dichotomized by the occurrence of PSE

	Total cohort (n = 348; 100%)	PSE (n = 32; 9.2%)	No PSE (n = 316; 90.8%)	*p*-value
Age, years, median (range)	67.00 (18–89)	59.50 (30–78)	67.00 (18–89)	0.095
Sex, n (%)				0.229
Male	193 (55.0)	21 (65.6)	172 (54.4)	
Female	155 (45.0)	11 (34.4)	144 (45.6)	
NIHSS at admission, median (range)	15 (0–42)	14 (4–42)	14 (0–42)	0.124
NIHSS postthrombectomy, median (range)	5 (0–42)	5 (0–18)	5 (0–42)	0.717
Vascular risk factors, n (%)				
Arterial hypertension	255 (73.28)	23 (71.88)	232 (73.42)	0.837
Dyslipidemia	190 (54.60)	16 (0.50)	174 (55.06)	0.600
Atrial fibrillation	146 (41.95)	9 (28.13)	137 (43.35)	0.108
Diabetes mellitus	54 (15.52)	2 (6.25)	52 (16.46)	0.147
Chronic alcohol abuse	15 (4.31)	1 (3.13)	14 (4.43)	0.728
Smoking	60 (17.24)	6 (18.75)	54 (17.09)	0.784
TICI score, n (%)				0.607
0–2a	26 (7.5)	4 (12.5)	22 (7.0)	
2b-3	322 (92.5)	28 (87.5)	294 (93.0)	
IV thrombolysis, n (%)	241 (69.3)	21 (65.6)	220 (69.6)	0.572
Etiology, n (%)				0.231
Large-artery atherosclerosis	75 (21.6)	6 (18.8)	69 (21.8)	0.703
Cardioembolic	163 (46.8)	12 (37.5)	151 (47.8)	0.272
Other/unknown	110 (31.6)	14 (40.0)	96 (30.4)	0.131
Early seizures, n (%)	14 (4.0)	3 (9.4)	11 (3.5)	0.090
SeLECT score post-thrombectomy, median (range)	4.0 (0–8)	4.0 (1–8)	4.0 (0–8)	0.682
Symptom onset to recanalization time, median hh:mm (range)	04:25 (01:18–21:42)	03:54 (02:15–13:52)	04:30 (01:18–21:42)	0.482
Follow up time (months), median (range)	77.6 (43–127)	88.0 (43–127)	76.6 (43–127)	0.252

PSE = poststroke epilepsy, NIHSS = National Institutes of Health Stroke Scale, TICI = Thrombolysis in Cerebral Infarction

**Table 2:** Neuroimaging features (on postthrombectomy MRI) in relation to the development of PSE

	Total cohort (n = 348; 100%)	PSE (n = 32; 9.2%)	No PSE (n = 316; 90.8%)	*p*-value
Affected cerebrovascular territory, n (%)				
Anterior cerebral artery	32 (9.20)	6 (18.75)	26 (8.23)	0.062
Middle cerebral artery	294 (84.48)	30 (93.75)	264 (83.54)	0.152
Posterior cerebral artery	75 (21.55)	5 (15.63)	70 (22.15)	0.427
Anterior choroidal artery	11 (3.16)	0	11 (3.48)	0.492
Vertebrobasilar arteries	65 (18.68)	5 (15.63)	60 (18.99)	0.638
Infarct location, n (%)				
Frontal	235 (67.53)	27 (84.38)	208 (65.82)	0.042
Temporal	250 (71.84)	28 (87.50)	222 (70.25)	0.049
Mesiotemporal	102 (29.31)	12 (37.50)	90 (28.48)	0.272
Parietal	210 (60.34)	27 (84.38)	183 (57.91)	0.007
Occipital	83 (23.85)	7 (21.88)	76 (24.05)	0.808
Thalamus	38 (10.92)	4 (12.50)	34 (10.76)	0.728
Basal ganglia	252 (72.41)	23 (71.88)	229 (72.47)	0.946
Brainstem	45 (12.93)	2 (6.25)	43 (13.61)	0.254
Cerebellum	59 (16.95)	4 (12.50)	55 (17.41)	0.474
Cortical involvement, n (%)	316 (90.8)	31 (96.9)	285 (90.2)	0.236
Infarct size, n (%)				
< 1/3 of territory	251 (72.1)	13 (40.6)	238 (75.3)	0.00009
1–2/3 of territory	75 (21.6)	13 (40.6)	62 (19.6)	0.007
> 2/3 of territory	22 (6.3)	6 (18.8)	16 (5.1)	0.003
Side of infarction, n (%)				0.060
Left	142 (40.8)	17 (53.1)	125 (39.6)	
Right	131 (37.6)	12 (37.5)	119 (37.7)	
Bilateral	75 (21.6)	3 (9.4)	72 (22.8)	
Microbleeds present, n (%)	51 (14.7)	9 (28.1)	42 (13.3)	0.021
Microbleeds presence and location, n (%)				0.072
Lobar	46 (13.2)	9 (28.1)	37 (11.7)	
Deep (basal ganglia/thalamus)	5 (1.4)	0	5 (1.6)	
Any intracranial bleeding, n (%)	136 (39.1)	14 (43.6)	122 (38.6)	0.603
Intracranial bleeding type, n (%)				
Hemorrhagic transformation I°	52 (14.94)	5 (15.63)	47 (14.87)	0.891
Hemorrhagic transformation II°	54 (15.52)	7 (21.88)	47 (14.87)	0.296
Parenchymal hematoma I°	23 (6.61)	1 (3.13)	22 (6.96)	0.478
Parenchymal hematoma II°	7 (2.01)	1 (3.13)	6 (1.90)	0.610
Previous infarcts, n (%)	96 (27.6)	6 (18.8)	90 (28.5)	0.462
Cortical	38 (10.9)	5 (15.6)	33 (10.4)	0.350
Lacunar	58 (16.7)	1 (3.1)	57 (16.4)	0.068
WMH (Fazekas score), median; n (%)	1	1	1	0.717
0 (none)	93 (26.7)	13 (40.6)	80 (25.3)	
1 (mild)	188 (54.0)	12 (37.5)	176 (55.7)	
2 (moderate)	46 (13.2)	3 (9.4)	43 (13.6)	
3 (severe)	21 (6.0)	4 (12.5)	17 (5.4)	
Ventricular atrophy score, median (IQR)	3 (1–9)	3 (1–7)	3 (1–9)	0.951
Cerebral atrophy score, median (IQR)	3 (1–9)	3 (1–7)	3 (1–9)	0.838

IQR = interquartile range, WMH = white matter hyperintensities

While MT patients who did not receive postinterventional brain MRI (and thus were excluded from this study) were older and had higher NIHSS scores, there were no significant differences in sex and IV thrombolysis rates (Supplemental Table S2).

In this cohort, 14 patients (4%) had early seizures and 32 patients (9%) developed PSE. The median time between the index stroke and the first early seizure was 1.5 days (range 0–5) and 477 days (range 9–2577 days) until first late seizure (PSE diagnosis).

The main seizure types according to the ILAE classification of 2017 in early and late seizures both were ‘focal to bilateral tonic–clonic’ > ‘focal onset, aware, motor onset’ (Supplemental Table S3).

In all cases, early seizures were treated with anti-seizure medication and, unless no further seizure occurred, treatment was discontinued after several weeks. Three patients with early seizures developed PSE and needed restarting of antiseizure medication.

In univariable Cox regression, patients who developed PSE had larger infarct sizes and more often lesions in the parietal, frontal and temporal lobes than patients without PSE. The presence of microbleeds (and their number) were also more prevalent respectively higher in PSE patients.

Occurrence of PSE was not related to initial or postinterventional NIHSS, stroke etiology (large-artery disease, cardioembolic, other/unknown), IV thrombolysis, common vascular risk factors, SeLECT score, or other neuroimaging characteristics on brain MRI (Tables 1, 2).

In multivariable logistic regression analysis (Table 3), larger infarct size (> 1/3 of affected cerebrovascular territory) and presence of microbleeds remained independently associated with PSE (area under the receiver operating characteristic curve = 0.75; 95% CI 0.66–0.83).

**Table 3:** Multivariable Cox regression analysis with occurrence of PSE as the target variable

Multivariable analysis	Hazard ratios (95% CI)	*p*-value
Infarct size > 1/3 of affected territories	3.49 (1.67–7.30)	0.0009
Microbleeds present	2.56 (1.18–5.56)	0.018
Frontal lobe affected	0.84 (0.21–3.31)	0.802
Temporal lobe affected	1.26 (0.31–5.09)	0.742
Parietal lobe affected	2.54 (0.72–9.02)	0.149

## Discussion

In this cohort of patients with large vessel occlusion stroke receiving mechanical thrombectomy, 9% developed PSE within a median follow-up duration of 6.5 years. In comprehensive analyses on clinical and MRI-based features and over a considerably long-term follow-up period, we identified larger infarct size and presence of microbleeds on postinterventional brain MRI as independent risk factors for PSE occurrence. Notably, the recently proposed and widely-used SELECT score [5] was not able to indicate PSE risk in this cohort.

Patients with larger infarct size on postinterventional brain MRI exposed an increased risk for PSE, which is not surprising and has also been reported in CT-based studies in general stroke patients and in those that had received MT [6, 21–23]. Moreover, neither recanalization status (TICI score) nor additional intravenous thrombolysis therapy was associated with PSE, in accordance with previous studies [18, 24, 25]. However, in this context, it needs to be emphasized that the rate of successful recanalization (in the two investigated high-volume stroke centers) was high with 92.5%, substantially limiting the size of the comparison group.

The observed increased rates of PSE in patients with infarction in the parietal, frontal and temporal lobes in univariable analyses were not confirmed in the multivariable analysis, which might be explained by the overall larger infarct size in PSE patients (with more different brain lobes affected).

One recent study found an elevated PSE risk after infarction in the right superior frontal cortex and the right frontal operculum [14]. Limitations of this study were the small number of patients (n = 132) who were generally older (mean non-PSE: 77 years, mean PSE: 75 years), the exclusion of patients with hemorrhagic transformation as well as those without brain MRI within 30 days after stroke and the short follow-up time of 1 year.

In our comprehensive analysis on acute and chronic cerebrovascular or other pre-existing parenchymal abnormalities on brain MRI, we found that microbleeds constituted a previously underrecognized risk feature for PSE. Information on the presence and distribution of cerebral microbleeds in association with epileptic seizures is scarce, especially in ischemic stroke patients. One previous study found a correlation between brain microbleeds and late seizures in patients with intracranial hemorrhage and suggested a link with underlying cerebral amyloid angiopathy (CAA) [26]. An association with CAA and PSE is unlikely in our cohort of MT patients, further supported by the fact that we did not identify any patient fulfilling the Boston MRI criteria for probable CAA [27]. Delayed epileptic seizures in (lobar) microbleeds might be caused by cortical irritation from hemosiderin depositions and gliotic scarring as well as inflammatory processes involved in epileptogenesis [7, 28]. In this context, it was interesting that we did not find a different effect between lobar and deep microbleeds and that hemorrhagic transformation was not associated with PSE as it has also been described in previous studies [29, 30].

Interestingly, WMH as well as ventricular and cerebral atrophy were not associated with PSE occurrence in our cohort. Previous studies mostly did not analyze the potential association between chronic microangiopathic lesions or atrophy in relation to epilepsy risk in stroke patients. One CT-based study found PSE less frequent in patients with leukoaraiosis [13].

We observed about three-quarter of the PSE incidence predicted by the SeLECT score (12% within 60 months for a median post-TE SeLECT score of 4 points) [5]. One potential reason for this might be the high rate of successfully recanalized arteries (TICI 2b-3: 92.5%) leading to lower sizes of ischemic infarcts in our study. This may be explained by the fact that temporary (more severe) ischemic brain injury exerts little effect on the occurrence of poststroke seizures and permanent structural damage of certain brain networks is underlying the process of epileptogenesis. Only few studies showed a positive correlation between the effect of recanalization therapy and PSE [16], but most did not, [25, 31, 32] supporting our hypothesis of relation of permanent ischemic brain damage and PSE.

When interpreting the findings of this study, some potential limitations have to be considered. First, we chose a retrospective study design to guarantee a sufficiently long period of follow up to detect a significant number of PSE. Second, as we aimed for a detailed analysis of both acute and chronic neuroimaging markers in relation to PSE risk, we only considered patients with a post-thrombectomy brain MRI. Third, the follow up strategies in the two stroke centers Linz and Graz were different. However, the rate of PSE occurrence was comparable (Linz: 10.6%, Graz: 8.4%). Finally, we only included patients after endovascular stroke treatment and thus findings do not reflect general ischemic stroke patients.

### Supplementary Information

Below is the link to the electronic supplementary material.

Supplementary file 1 (DOCX 21 KB)
