# Supplementary material for: Frequency and predictors of poststroke epilepsy after mechanical thrombectomy for large vessel occlusion stroke: results from a multicenter cohort study
Source: J Neurol. 2023 Sep 2;270(12):6064–70. doi: 10.1007/s00415-023-11966-x (PMC10632247; doi:10.1007/s00415-023-11966-x)
Supplement: Supplementary file 1 — (DOCX 21 KB) [file 415_2023_11966_MOESM1_ESM.docx]

**Supplemental tables**

**Frequency and predictors of poststroke epilepsy after mechanical thrombectomy for large vessel occlusion stroke: Results from a multicenter cohort study**

*Joachim Gruber (MD)^1^; Thomas Gattringer (MD, PhD)^2,3#^; Georg Mayr^4^ (MD); Daniel Schwarzenhofer^1^ (MD); Markus Kneihsl^2,3^ (MD, PhD); Judith Wagner^6^ (MD); Michael Sonnberger^4^ (MD); Hannes Deutschmann^5^ (MD); Melanie Haidegger^2^ (MD); Simon Fandler-Höfler (MD, PhD)^2^; Stefan Ropele^2^ (MD); Christian Enzinger^2*^ (MD); Tim von Oertzen^1#*^(MD)*

1 Department of Neurology 1, Neuromed Campus, Kepler University Hospital, Linz, Austria

2 Department of Neurology, Medical University of Graz, Graz, Austria

3 Division of Neuroradiology, Vascular and Interventional Radiology, Department of Radiology, Medical University of Graz, Graz, Austria

4 Department of Neuroradiology, Neuromed Campus, Kepler University Hospital, Linz, Austria

5 Division of Neuroradiology, Vascular and Interventional Radiology, Department of Radiology, Medical University of Graz, Graz, Austria

6 Department of Neurology, Evangelisches Klinikum Gelsenkirchen, Academic Hospital University Essen-Duisburg, Gelsenkirchen, Germany

**^#^Correspondence:**

Dr. Thomas Gattringer

Department of Neurology, Medical University of Graz

Auenbruggerplatz 22, 8026 Graz, Austria

Tel: 0043 316 385 80231

Mail: thomas.gattringer@medunigraz.at

Dr. Tim von Oertzen

Department of Neurology 1, Neuromed Campus, Kepler University Hospital, Linz, Austria

Wagner-Jauregg-Weg 15, 4020 Linz, Austria

Mail: tim.vonoerzten@kepleruniklinikum.at

*Contributed equally as senior authors

Supplemental table 1: overview of collected clinical and imaging data

| Clinical variables | Imaging variables |
| --- | --- |
| Age at stroke onset (years) | Imaging quality (poor, intermediate, good) |
| Sex (m/f) | Side of infarction (left/right/both) |
| Onset and time of stroke (date, time) | Involved vessel territory (ICA, ACA, MCA, PCA, BA, AChA) |
| Severity of stroke measured by NIHSS score (1-42) | Size of affected vessel territory (< 1/3, > 1/3 – 2/3, > 2/3) |
| SeLECT Score before and after thrombectomy (1-9) | Cortical or subcortical affection |
| Etiology according to TOAST criteria (Adams HP Jr et al., 1993) | Type of infarction (territorial, watershed, multiembolic) |
| Intravenous bridging lysis therapy (yes/no) | Intracranial bleeding by ECASS classification (del Zoppo GJ. Et al., 1992) |
| Grade of recanalization through mechanical thrombectomy measured by the TICI score (Higashida RT et al., 2003) | White matter hyperintensities (WMH) by Fazekas classification (Fazekas F. et al., 1987) |
| Occurrence of early and late seizures (yes/no) | Number and localization of microbleeds (≤ 5 mm) |
| Onset and frequency of seizures (date, grading) | Number and localization of previous infarcts (cortical or lacunar) |
| Type(s) of seizure(s) according to ILAE 2017 classification (Fisher RS et al., 2017) | Cerebral atrophy by SIFAP classification |
| Anti-seizure medication and dose | Ventricular atrophy by SIFAP classification |
| Response to anti-seizure medication (poor, intermediate, good, unknown) | Other brain lesions |
| Cerebrovascular risk factors (arterial hypertension, dyslipidemia, atrial fibrillation, diabetes mellitus, harmful alcohol abuse, chronic nicotine abuse |  |

|  | **CT (n = 704)** | **MRI (n = 348)** | **Total (n = 1052)** | **P-value** |
| --- | --- | --- | --- | --- |
| Age, median (range) | 72  (27 - 93) | 67  (18 - 89) | 71  (18 - 93) | < 0.00001 |
| Sex, n (%)  Male  Female | 391 (55.5%)  313 (44.5%) | 194 (55.7%)  155 (44.3%) | 585 (55.6%)  468 (44.4%) | 0.980 |
| NIHSS, median (range) | 16 (1 - 42) | 14 (0 - 42) | 15 (0 - 42) | < 0.00001 |
| IV thrombolysis, n (%) | 473 (67.2%) | 241 (69.3%) | 714 (67.8%) | 0.520 |

Supplemental table 2: characteristics of patient groups with CT and MRI scan

Supplemental table 3: seizure characteristics of patient with ES and PSE

|  | **Early seizures (n = 14)** | **Poststroke epilepsy (n = 32)** |
| --- | --- | --- |
| Time until first seizure, days median (min.-max.) | 1,5 (0 – 5) | 477 (9 – 2577) |
| Seizure types |  |  |
| Focal onset, aware, motor onset; n (%) | 3 (21.4) | 9 (28.1) |
| Focal to bilateral tonic-clonic; n (%) | 4 (28.6) | 16 (50) |
| Focal onset, aware, non-motor onset; n (%) | 0 | 3 (9.4) |
| Focal onset, impaired awareness, non-motor onset; n (%) | 2 (14.3) | 2 (6.3) |
| Status epilepticus, non-motor; n (%) | 0 | 3 (9.4) |
| Status epilepticus, motor; n (%) | 0 | 3 (9.4) |
| Focal onset, impaired awareness, motor onset; n (%) | 2 (14.3) | 1 (3.1) |
| Unknown onset, tonic-clonic; n (%) | 2 (14.3) | 0 |
| Unknown onset, other motor; n (%) | 0 | 1 (3.1) |
| Unknown onset, unclassified; n (%) | 1 (7.1) | 1 (3.1) |
| Seizure frequency  once; n (%)  < 1 seizure per year; n (%)  < 2 seizures per year; n (%)  2 – 4 seizures per year; n (%)  5 – 12 seizures per year; n (%)  > 1 seizure per month; n (%)  > 1 seizure per week; n (%)  unknown; n (%) | 14 (100)  -  -  -  -  -  -  - | 12 (37.5)  6 (18.8)  5 (15.6)  1 (3.1)  1 (3.1)  0  1 (3.1)  4 (12.5) |
| Anti-seizure medication (ASM)^1^, first  Levetiracetam (LEV); n (%)  Lamotrigine (LTG); n (%)  Lacosamide (LCS); n (%)  Oxcarbazepine (OCX); n (%)  None; n (%) | 10 (71.4)  0  4 (28.6)  0  0 | 23 (71.9)  3 (9.4)  2 (6.3)  1 (3.1)  3 (9.4) |
| Anti-seizure medication (ASM), second  Lacosamide (LCS); n (%)  Oxcarbazepine (OXC); n (%) | 0  0 | 1 (3.1)  1 (3.1) |
| ASM effect  poor (no effect); n (%)  intermediate (> 50% reduction); n (%)  good (seizure free); n (%)  unknown | 0  0  4 (28.6)  0 | 0  5 (15.6)  26 (81.3)  1 (3.1) |

^1^ in all ES cases treatment was discontinued after several weeks
